# Genetic variants in the *MRPS30 *region and postmenopausal breast cancer risk

**DOI:** 10.1186/gm258

**Published:** 2011-06-24

**Authors:** Ying Huang, Dennis G Ballinger, James Y Dai, Ulrike Peters, David A Hinds, David R Cox, Erica Beilharz, Rowan T Chlebowski, Jacques E Rossouw, Anne McTiernan, Thomas Rohan, Ross L Prentice

**Affiliations:** 1 Fred Hutchinson Cancer Research Center, Divisions of Public Health Sciences, and Vaccine and Infectious Diseases, 1100 Fairview Avenue North, Seattle, WA 98109-1024, USA; 2 Perlegen Sciences Inc., 2021 Stierlin Court, Mountain View, CA 94043, USA; 3 23andMe, Inc., 1390 Shorebird Way, Mountain View, CA 94043, USA; 4 Harbor-UCLA Research and Education Institute, Division of Medical Oncology/Hematology, 1124 W. Carson Street, Bldg J-3, Torrance, CA 90502-2064, USA; 5 National Institutes of Health, National Heart, Lung and Blood Institute, Prevention and Population Sciences Program, 6701 Rockledge Drive, Bethesda, MD 20892-7935, USA; 6 Albert Einstein College of Medicine, Department of Epidemiology and Population Health, 1300 Morris Park Avenue, Bronx, NY 10461, USA

## Abstract

**Background:**

Genome-wide association studies have identified several genomic regions that are associated with breast cancer risk, but these provide an explanation for only a small fraction of familial breast cancer aggregation. Genotype by environment interactions may contribute further to such explanation, and may help to refine the genomic regions of interest.

**Methods:**

We examined genotypes for 4,988 SNPs, selected from recent genome-wide studies, and four randomized hormonal and dietary interventions among 2,166 women who developed invasive breast cancer during the intervention phase of the Women's Health Initiative (WHI) clinical trial (1993 to 2005), and one-to-one matched controls. These SNPs derive from 3,224 genomic regions having pairwise squared correlation (r^2^) between adjacent regions less than 0.2. Breast cancer and SNP associations were identified using a test statistic that combined evidence of overall association with evidence for SNPs by intervention interaction.

**Results:**

The combined 'main effect' and interaction test led to a focus on two genomic regions, the fibroblast growth factor receptor two (*FGFR2*) and the mitochondrial ribosomal protein S30 (*MRPS30*) regions. The ranking of SNPs by significance level, based on this combined test, was rather different from that based on the main effect alone, and drew attention to the vicinities of rs3750817 in *FGFR2 *and rs7705343 in *MRPS30*. Specifically, rs7705343 was included with several *FGFR2 *SNPs in a group of SNPs having an estimated false discovery rate < 0.05. In further analyses, there were suggestions (nominal *P *< 0.05) that hormonal and dietary intervention hazard ratios varied with the number of minor alleles of rs7705343.

**Conclusions:**

Genotype by environment interaction information may help to define genomic regions relevant to disease risk. Combined main effect and intervention interaction analyses raise novel hypotheses concerning the *MRPS30 *genomic region and the effects of hormonal and dietary exposures on postmenopausal breast cancer risk.

## Background

Genome-wide association studies have identified a substantial number of common genetic variants that are associated with risk, for each of several diseases. However, most such associations are weak and account for only a small fraction of familial disease aggregation [1]. In the case of breast cancer, seven reproducible genetic susceptibility alleles were estimated to explain about 5% of heritability [2]. Studies of low frequency genetic variants, gene-gene interactions, genotype by environment interaction, and shared environment have been suggested [1] as means to identify the 'missing heritability' for complex diseases, along with more thorough study of variants within genomic regions of interest.

Closely related to this is the role of genetic variants in model discrimination and disease risk prediction. A recent multiple-cohort analysis of ten common genetic variants that reliably associate with breast cancer concluded that 'the level of predicted breast cancer risk among most women changed little' when these SNPs were added to existing risk assessment models [3]. In response, an accompanying editorial [4] pointed out that cellular networks within which the SNPs operate may associate more strongly with risk than do tagging SNPs alone, that gene-gene and gene-environment interactions are 'likely to be profoundly important', and that associations with breast cancer subtypes may be more impressive.

A challenge to pursuing the gene-environment concept is the typical difficulty in assessing key environmental exposures. For example, given the well-established association between obesity and post-menopausal breast cancer risk, one might expect that total energy consumption and other dietary factors may influence breast cancer risk, possibly in a manner that depends on genetic factors that relate to hormone metabolism, growth factors, or inflammation. However, dietary data are attended by random and systematic assessment biases that may seriously attenuate and distort estimated associations [5].

Randomized controlled intervention trials can provide highly desirable settings for the incorporation of genotype by environment interactions into genetic association analyses. First, the intervention group assignment is known with precision, and secondly, this assignment is statistically independent of underlying genotype by virtue of randomization. This latter feature also allows highly efficient case-only test statistics [6-8] to be used for genotype by intervention interaction testing.

The Women's Health Initiative (WHI) randomized controlled trial included four randomized and controlled comparisons among postmenopausal women in a partial factorial design [9,10]. Specifically, it comprised a postmenopausal hormone therapy component that involved two non-overlapping trials: estrogen versus placebo (E-alone trial) among women who were post-hysterectomy, and estrogen plus progestin versus placebo (E+P trial) among women with a uterus; a low-fat dietary modification (DM) versus usual diet component, and a calcium and vitamin D (CaD) versus placebo supplementation component.

An elevation of breast cancer risk triggered the early stopping of the E+P trial in 2002 [11,12]. In the E-alone trial, which was stopped early in 2004 primarily due to an elevation of stroke risk [13], there was a surprising suggestion of a reduction in breast cancer risk in the intervention group, as well as apparent interactions of the E-alone hazard ratio with several other breast cancer risk factors [14]. The DM trial continued to its planned termination in 2005. While overall it provided non-significant evidence of a breast cancer reduction over its 8.1-year average follow-up period, the breast cancer hazard ratio was significantly lower in the quartile of women who had a comparatively high fat content in their diet at baseline [15]. These women made a larger dietary change if assigned to the low-fat diet intervention. The CaD trial did not yield evidence of an effect on breast cancer risk [16].

We studied 4,988 SNPs in relation to breast cancer incidence and clinical trials intervention effects during the intervention phase of the WHI clinical trial. Nearly all of these SNPs were selected as the top-ranked SNPs according to significance level for association with breast cancer in the NCI Cancer Genetic Markers of Susceptibility (C-GEMS) genome-wide association study [17], while the remaining 244 were selected based on published data from the Breast Cancer Association Consortium genome-wide association study [18]. These SNPs were scattered throughout the genome. In fact, they arise from 3,224 distinct loci when a squared pairwise correlation (r^2^) between adjacent regions of less than 0.2 is used to define new loci. We ranked SNPs according to a null hypothesis test that combined evidence of overall breast cancer association with evidence of interaction with one or more of the randomized clinical trial intervention assignments.

## Materials and methods

### Study design and population

Enrollees in WHI trials were postmenopausal women aged 50 to 79 years who met component-specific eligibility criteria [19]. Women were randomized to a hormone therapy component, or a DM component, or both. At the one-year anniversary from enrollment, participating women could be further randomized into a CaD supplementation component. A total of 68,132 women were enrolled into the trials between 1993 and 1998, among which there were 10,739 in E-alone, 16,608 in E+P, 48,835 in DM, and 36,282 in CaD components. Details about distributions of demographic variables and breast cancer risk factors in the study cohort were published previously [19]. For the DM trial we chose to focus interaction testing on the subset of 12,208 women having baseline percentage of energy from fat in the upper quartile, and we denote the DM intervention in this sub-cohort by DMQ.

### Case and control selection

All 2,242 invasive breast cancer cases that developed between randomization and the end of the trial intervention phase (31 March 2005) were considered for inclusion, among which a total of 2,166 (96.6%) cases had adequate quantity and quality of DNA. This leads to analyses based on 247 cases for E-alone, 471 cases for E+P, 428 cases for DMQ, 1,049 cases for CaD (cases arising after CaD randomization only), and corresponding controls that were one-to-one matched to cases on baseline age, self-reported ethnicity, participation in each trial component, years since randomization, and baseline hysterectomy status.

### Laboratory methods

Genotyping and data cleaning methods at Perlegen Sciences (Mountain View, CA, USA) have been described [20]. The average call rate for these SNPs was 99.8%, and the average concordance rate for 157 blind duplicate samples was also 99.8%.

Principal component analysis was used to characterize population structure and to identify genotyping artifacts. The top 20 principal components did not associate with common sources of experimental variability (for example, date of sample processing or hybridization performance for either chip design). The first ten principal components were found to account for 86% of the total SNP genotype variation, while the first four principal components provided good separation among the major self-reported 'ethnicities' (white, black, Hispanic, Asian/Pacific Islander, northern versus southern European ancestry).

### Statistical methods

A five-component test statistic was used for each SNP to test association with breast cancer. The first 'main effect' component arose as score test from a standard logistic regression of case (1) versus control (0) status on number of minor SNP alleles and potential confounding factors. The logistic regression model included the (log transformed) Gail 5-year breast cancer risk score [21], previous hormone use (indicators for < 5, 5 to 10, and ≥10 years for each of estrogen and estrogen plus progestin), and (log transformed) body mass index. Also included are variables used for matching controls to cases in control selection. In addition, eigenvectors from the first ten principal components from correlation analysis of the genotype data were included to adjust for population stratification [22]. The other four test statistic components were case-only tests for dependence of intervention odds ratios on SNP genotype for each of E-alone, E+P, DMQ, and CaD. These statistics arise as score tests in logistic regression of active (1) versus placebo or usual diet (0) randomization assignment on the number of minor SNP alleles with logistic regression location parameter offset by log q/(1 - q), where q is the fraction of women assigned to active intervention for the pertinent clinical trial component. The main effect test statistic is asymptotically independent of each of the case-only test statistics [23], and the interaction tests for E-alone and E+P are independent since they are based on non-overlapping sets of women. A 'sandwich' variance estimator was used to allow for possible correlations among the other pairs of case-only test statistics. A chi-square test with five degrees of freedom was then used to test SNP association with breast cancer, for each of the SNPs. Further details about this joint test procedure are included here as Additional file 1.

SNPs of interest in these association tests were subsequently examined for evidence of main effect and interaction effects separately. The latter once again employed case-only analyses, and for descriptive purposes, intervention odds ratios were estimated separately at zero, one, and two minor SNP alleles. A likelihood ratio test with two degrees of freedom assessed SNP by intervention interaction in these analyses.

The potential of SNP by clinical trial interactions to contribute to the ability to discriminate between breast cancer cases and controls was evaluated by estimating areas under the receiver operating characteristic curves (AUC), and associated confidence intervals.

Some further analyses were carried out with breast cancers classified according to either the estrogen receptor status or the progesterone receptor status of the breast tumor. All significance levels (*P*-values) are two-sided.

### Ethics approval

This research conforms to the Helsinki Declaration and pertinent legislation, and has been approved by the Institutional Review Board of the Fred Hutchinson Cancer Research Center. All women included in this report provided informed consent that permitted their biospecimens and data to be used in the present research project.

## Results

### Simultaneous tests of main effect and interaction with clinical trial interventions

Table 1 presents the top 20 SNPs ranked by *P*-value of the combined test of main effect and interaction. Among the 4,988 SNPs evaluated, six SNPs have the joint test *P*-value less than 10^-6 ^and a false discovery rate (FDR) less than 0.0005, all in the *FGFR2 *(fibroblast growth factor receptor 2) region in chromosome region 10q16. Immediately following are several SNPs from the *MRPS30 *(mitochondrial ribosomal protein S30) region in chromosome region 5p12. Of these SNPs, rs7705343 is included in the set of SNPs having FDR < 0.05, while close-by SNP rs13159598 is also among SNPs having FDR < 0.10.

**Table 1:** Top 20 SNPs identified by combined test for main effect and interaction with clinical trial interventions

Rank^a^	Rs number^b^	Chromosome	Position	MAF^c^	Allele^d^	Combined test *P*-value^e^	Combined test FDR^f^	Main effect test *P*-value^g^	Main effect test rank^h^	Gene
1	rs1219648	10q26	123336180	0.42	G/A	6.45E-09	3.21E-05	3.90E-10	1	*FGFR2*
2	rs2981579	10q26	123327325	0.44	A/G	7.76E-09	1.94E-05	2.78E-09	2	*FGFR2*
3	rs3750817	10q26	123322567	0.37	T/C	5.61E-08	9.32E-05	9.02E-08	5	*FGFR2*
4	rs11200014	10q26	123324920	0.41	A/G	1.08E-07	0.000135	3.40E-09	3	*FGFR2*
5	rs2420946	10q26	123341314	0.42	T/C	1.56E-07	0.000156	1.49E-08	4	*FGFR2*
6	rs2981582	10q26	123342307	0.41	A/G	5.25E-07	0.000437	9.99E-08	6	*FGFR2*
7	rs7705343	5p12	44915334	0.42	G/A	5.88E-05	0.0419	0.000355	11	*MRPS30*
8	rs13159598	5p12	44841683	0.42	G/A	0.000136	0.0846	0.000425	13	*MRPS30*
9	rs11746980	5p12	44935642	0.43	C/T	0.000240	0.133	0.000511	16	*MRPS30*
10	rs9790879	5p12	44813635	0.43	A/G	0.000244	0.122	0.000963	19	*MRPS30*
11	rs2330572	5p12	44776746	0.43	C/A	0.000294	0.133	0.00129	22	*MRPS30*
12	rs7555040	1p33	47641903	0.13	G/A	0.000336	0.140	0.002483	26	Unknown
13	rs4415084	5p12	44698272	0.43	T/C	0.000400	0.153	0.000436	14	*MRPS30*
14	rs994793	5p12	44779004	0.43	G/A	0.000417	0.148	0.00184	23	*MRPS30*
15	rs2218080	5p12	44750087	0.44	C/T	0.000446	0.148	0.00274	30	*MRPS30*
16	rs7795554	7p21	12159269	0.36	C/T	0.000498	0.155	0.00353	40	Unknown
17	rs7519783	1q32	198951680	0.27	G/A	0.000904	0.265	0.229	1160	Unknown
18	rs1499111	4q28	129691789	0.22	T/C	0.00115	0.318	0.0736	431	Unknown
19	rs719278	3q11	98887302	0.40	A/G	0.00122	0.320	0.238	1204	*EPHA6*
20	rs1232355	3q26	88073313	0.05	C/T	0.00132	0.329	0.179	942	Unknown

^a^Rank, rank of SNPs based on combined test *P*-value; ^b^Rs number, SNP identification (rs) number in dbSNP database; ^c^MAF, minor allele frequency in the study population; ^d^Allele, minor/major allele; ^e^Combined test *P*-value, *P*-value based on the simultaneous test with 5 df; ^f^Combined test FDR, FDR based on the simultaneous test with 5 df; ^g^Main effect *P*-value, *P*-value based on main effect test only; ^h^Main effect rank, rank of SNPs based on main effect *P*-value.

Table 1 also shows *P*-values and rankings for these SNPs under the main effect association test alone. While *P*-values for *FGFR2 *SNPs tend to be somewhat diluted by the inclusion of the interaction information in the test statistic, the ordering of these SNPs is rather different under the two-testing procedures. For example, SNP rs3750817, which is in a somewhat separate linkage disequilibrium bin from tagging SNP rs2981582 [18], has a comparatively higher ranking with the combined test. We have previously reported suggestive evidence of interaction of rs3750817 with E-alone and E+P [24], and DMQ [25].

**Figure 1:**
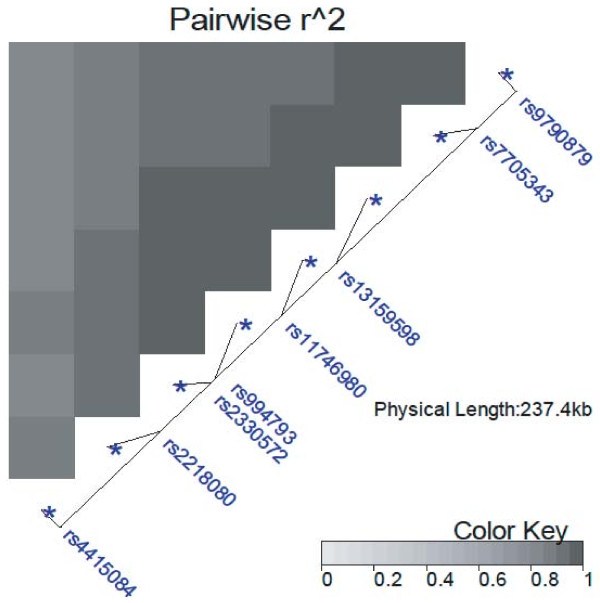
**Pairwise *r^2 ^*for SNPs within the *MRPS30 *region in chromosome 5p12, where *r *is the allelic correlation between SNPs**.

SNPs in the *MRPS30 *region of chromosome 5p12 have a higher ranking overall with the combined versus the main effect test. Moreover, the ordering of SNPs within this region is considerably altered by the inclusion of the interaction information. These analyses point to the genomic region in proximity of rs7705343 as relevant to breast cancer risk. Figure 1 shows squared pairwise correlations (r^2^) among SNPs in the *MRPS30 *region of chromosome 5p12. The combined test rankings tend to decrease as one moves from rs7705343 to the tagging SNP rs4415084 at the opposite end of this genomic region of approximately 230 kb.

Table 2 shows *P*-values individually for the five components of the combined test, for the eight SNPs in the *MRPS30 *region. Most of the association information derives from the main effect test, but the intervention interaction tests have rather different *P*-values across these SNPs, with rs7705343 having nominally significant (*P *< 0.05) interactions with each of E-alone, DMQ, and CaD, while interactions in relation to rs4415084 are not significant for any of the interventions.

**Table 2:** Significance levels (*P*-values) for testing interaction with WHI trial interventions for SNPs in the *MRPS30 *region

Rs number^a^	Chromosome	Position	Minor/major allele	MAF^b^	OR^c^	p.main^d^	E-alone^e^	E+P^f^	DMQ^g^	CaD^h^
7705343	5p12	44915334	G/A	0.40	1.18	0.000355	**0.043**	0.863	**0.042**	**0.046**
13159598	5p12	44841683	G/A	0.41	1.17	0.000425	0.056	0.920	0.057	**0.048**
11746980	5p12	44813635	A/G	0.41	1.16	0.000511	0.064	0.790	**0.043**	0.095
9790879	5p12	44935642	C/T	0.41	1.17	0.000963	0.117	0.762	**0.042**	**0.047**
2330572	5p12	44776746	C/A	0.42	1.16	0.00129	**0.042**	0.880	**0.043**	0.106
4415084	5p12	44698272	T/C	0.41	1.17	0.000436	0.242	0.944	0.127	0.146
994793	5p12	44779004	G/A	0.42	1.15	0.00184	0.084	0.798	**0.041**	0.080
2218080	5p12	44750087	C/T	0.43	1.15	0.00274	0.273	0.933	**0.025**	0.069

^a^Rs number, SNP identification (rs) number in dbSNP database; ^b^MAF, minor allele frequency in the study population; ^c^OR, estimated minor allele odds ratio under additive allelic effects model; ^d^p.main, significance level for SNP association with breast cancer in additive allele effects model; ^e^E-alone, *P-*value for dependence (interaction) of E-alone odds ratio on SNP from case-only analyses; ^f^E+P and ^h^CaD, corresponding interaction *P-*values for the other interventions; ^g^DMQ, interaction *P-*value for DM among women with baseline percentage energy from fat in the upper quartile. Entries in bold are interaction effects significant at the nominal (0.05) level. WHI, Women's Health Initiative.

Table 3 shows estimated intervention odds ratios and 95% confidence intervals as a function of the number of minor alleles of rs7705343 for each of the four interventions. The GG genotype is associated with lower intervention ORs for each of E-alone, DMQ, and CaD. Additional file 2 provides corresponding information with breast cancers classified according to estrogen receptor or progesterone receptor positivity. No clear variations by tumor receptor status were suggested, through statistical power for detecting moderate variations with tumor type is limited.

**Table 3:** Breast cancer odds ratio for WHI trial interventions by genotype of *MRPS30 *SNP rs7705343

		SNP genotype						
			
		GG		GA		AA		
					
Intervention	Number of cases	OR^a^	95% CI	OR^a^	95% CI	OR^a^	95% CI	*P*-value^b^
E-alone	247	0.484	(0.306, 0.766)	0.974	(0.684, 1.387)	0.969	(0.508, 1.846)	0.043
E+P	471	1.404	(1.003, 1.965)	1.248	(0.966, 1.613)	1.303	(0.858, 1.980)	0.863
DMQ	428	0.524	(0.360, 0.761)	0.862	(0.651, 1.141)	1.023	(0.643, 1.627)	0.042
CaD	1,049	0.763	(0.613, 0.951)	1.071	(0.902, 1.271)	1.049	(0.791, 1.391)	0.046

^a^OR, estimated intervention odds ratio; ^b^*P-*value, significance level for SNP interaction with clinical trial intervention. CI, confidence interval; WHI, Women's Health Initiative.

The majority (86%) of the case-control samples are from European-ancestry populations. In Additional files 3 and 4 we provide *P*-values for interaction between trial components and SNPs in the *MRPS30 *region, and the estimated intervention odds ratios and 95% confidence intervals as a function of the number of minor alleles of rs7705343 among women of European ancestry specifically. The patterns that we observe are quite similar to the overall patterns.

We also examined the joint associations of these *FGFR2 *and *MRPS30 *SNPs with hormonal and dietary intervention effects, using case-only analysis. Based on logistic regression applied to cases in DMQ, where the indicator for active treatment is regressed on genotypes of rs3750817 and rs7705343 together, both SNPs showed nominally significant interactions. The *P*-values for rs3750817 and rs7705343 were 0.0059 and 0.037. When E-alone was similarly considered, rs3750817 and rs7705343 had *P*-values of 0.053 and 0.043 in the joint interaction model.

The AUC was calculated from logistic regression analyses that included clinical trial randomization assignments for each of the four interventions and potential confounding factors. This gave an AUC (95% confidence interval) of 0.594 (0.578, 0.611). When main effect indicator variables were added for one and two minor alleles of rs3750817 and rs7705343, the AUC increased to 0.610 (0.594, 0.627). When SNP by intervention interaction indicator variables were also included, the AUC increased further to 0.621 (0.604, 0.637). A bootstrap test of significance for the genotype by intervention terms gave a nominal *P*-value of 0.007.

## Discussion

We evaluated the association between 4,988 SNPs and invasive breast cancer incidence in the WHI clinical trial through the use of a statistic that combines SNP main effect information with SNP by intervention interaction information for each of four randomized interventions. This view of the data provided a clear focus on two genomic regions, the *FGFR2 *region of chromosome 10 q, which has a very strong main effect along with suggestive evidence for interaction, and the *MRPS30 *region of chromosome 5 p, which shows evidence of a comparatively smaller main effect and suggestive evidence for interaction. The inclusion of the clinical trial interventions in this testing procedure leads to interest in subregions containing *FGFR2 *SNP rs3750817 and *MRPS30 *SNP rs7705343 that are some distance from their associated tagging SNPs, possibly suggesting more than one regulatory element in these non-coding genomic regions.

We have previously [9,10] discussed these data in relation to *FGFR2*. The eight *MRPS30 *SNPs considered here fall in a linkage disequilibrium region of approximately 230 kb from downstream of fibroblast growth factor 10 (*FGF10*) to downstream of *MRPS30*, with a minimum squared correlation among SNPs of 0.80 (Figure 1). *FGF10/FGFR2 *signaling [26-29] could be relevant to these associations, though there is a recombination hotspot between the *FGF10 *gene and the 5p12 SNPs studied here.

Our analyses suggest that interactions of these two SNPs with WHI clinical trial interventions lead to a detectable increase in the ability to distinguish breast cancer cases from controls. Note, however, that AUC values in this context may be optimistic in view of our procedure for identifying SNPs of interest. Moreover, since the interactions identified in the study have yet to be confirmed by replication studies, the increase in AUC detected here is of exploratory nature as well. Also note that AUCs estimated here tend to be somewhat low due to age matching in the case-control sample.

When our combined test is separated into its constituents, one observes nominally significant evidence of interaction of *MRPS30 *SNP rs7705343 with three of the four WHI interventions. Given the manner in which we ranked SNPs, these analyses (Tables 2 and 3) should be regarded as exploratory and such interactions will need to be confirmed separately. Unfortunately, other clinical trial data are not available for this purpose, and confirmation in observational study settings will involve the challenge of reliable ascertainment of the relevant hormonal or dietary exposures, and will need to be carried out in a case-control rather than case-only model. Hence, quite large numbers of cases and controls will be needed, as may be accessible through cohort consortia.

It is interesting to see a significant interaction of rs7705343 with E-alone with the estimated intervention OR below 1.0 for the GG genotype, and an insignificant interaction of rs7705343 with E+P with the estimated intervention OR greater than 1 for the GG genotype. Few interactions with study subject characteristics have been suggested for E+P [12], with *FGFR2 *SNP rs3750817 as a possible exception [24]. In contrast, interactions with several subject characteristics have been identified for E-alone, including family history of breast cancer, benign breast disease [14], and again *FGFR2 *SNP rs3750817 [24]. A possible explanation is that the progestin in E+P tends to overwhelm the minor variations in hormone therapy hazard ratios that would otherwise occur, giving rise to a strong and fairly uniform risk elevation.

Study strengths include its nesting within the randomized controlled WHI clinical trial, implying randomization assignments that are known and that are statistically independent of genotype and the related ability to use case-only analyses for intervention testing. Other strengths of the study include the use of pre-diagnostic blood specimens, collected and stored according to a standardized protocol, and quality-controlled SNP genotyping.

A limitation of the study is that the average age at enrollment was 63 years in the WHI controlled trials, with many women well past menopause at enrollment. We have reported, in combined clinical trials and observational studies analyses, higher breast cancer hazard ratios for E+P and E-alone among women who first use these preparations soon after the menopause, compared to those using them later [30,31]. Hence, the magnitude of the odds ratios shown here may be lower than would apply to typical hormone therapy users.

## Conclusions

Simultaneous consideration of overall association and intervention interaction point to genomic regions in the vicinity of *FGFR2 *and *MRPS30 *genes as relevant to breast cancer risk among postmenopausal women. Moreover, subregions that were not otherwise the focus of interest, in the vicinity of SNPs rs3750817 and rs7705343, were identified as worthy of further study by virtue of suggestive interactions with hormonal and dietary interventions. These analyses represent an early step in assessing the role of genotype by 'environment' interactions to help explain familial breast cancer patterns, or as a contributor to risk discrimination.

## Abbreviations

AUC: area under the receiver operating characteristic curve; CaD trial: calcium and vitamin D versus placebo supplementation component; DM trial: low-fat dietary modification versus usual diet component; DMQ: low-fat dietary modification trial in the subset of women having baseline percentage of energy from fat in the upper quartile; E-alone trial: estrogen versus placebo; E+P trial: estrogen plus progestin versus placebo; FDR: false discovery rate; *FGF10*: fibroblast growth factor 10; *FGFR2*: fibroblast growth factor receptor 2; *MRPS30*: mitochondrial ribosomal protein S30; SNP: single nucleotide polymorphism; WHI: Women's Health Initiative.

## Competing interests

RTC reports receiving consulting fees from AstraZeneca, Novartis, Pfizer, and Eli Lilly, lecture fees from AstraZeneca and Novartis, and grant support from Amgen. No other potential conflict of interest relevant to this article was reported.

## Authors' contributions

All authors were involved in development and/or critical review and revision of the manuscript. Additionally, DB, DH, DC, and EB had primary responsibility for project genotyping; YH, DH and RP had primary responsibility for data analysis; RC, JR, AM, TR and RP had responsibility for clinical data; and DB, UP and RP had primary administrative responsibility for this research project.

## Supplementary Material

Additional file 1 **Joint test of main and interaction effects**. Click here for file

Additional file 2 **Table S1**. Odds ratios for four clinical trial interventions by genotype of rs7705343 in the *MRPS30 *region according to tumor receptor status. Click here for file

Additional file 3 **Table S2**. Significance levels (*P*-values) for testing interaction with WHI trial interventions among women with European ancestry for SNPs in the *MRPS30 *region. Click here for file

Additional file 4 **Table S3**. Breast cancer odds ratio for WHI trial interventions among women of European ancestry by genotype of *the MRPS30 *SNP rs7705343. Click here for file
