# Supplementary material for: Genetic variants in the MRPS30 region and postmenopausal breast cancer risk
Source: Genome Med. 2011 Jun 24;3(6):42. doi: 10.1186/gm258 (PMC3218816; doi:10.1186/gm258)
Supplement: Additional file 1 — Joint test of main and interaction effects. [file gm258-S1.DOC]

**Additional file 1: Joint test of main and interaction effects**

Let Y = 0,1 indicate control and case status, respectively; let X be the vector of covariates including treatment assignment in the WHI trials, potential confounders (log transformed Gail 5-year breast cancer risk score, previous hormone use for each of estrogen and estrogen plus progestin, log transformed body mass index), variables used for matching controls to cases (baseline age, self-reported ethnicity, participation in each trial component, years since randomization, and baseline hysterectomy status), and first ten principal components for the SNP genotype data.

For a particular SNP, let G = 0,1,2 indicate the number of minor alleles.

The following five logistic regression models are applied to case-control or case-only samples:

(0) Applying a logistic regression model of Y on G and X to the case-control sample:

and let be the estimator of .

(1 to 4) Let Z_k_ = 0,1; k = 1,2,3,4 as the indicator that a subject is assigned to the placebo or active arm of the E-alone trial, E+P trial, DMQ trial, and CaD trial respectively. Applying a logistic regression model to all the cases in the corresponding trial component:

where is the offset term, with q_k_ the fraction of women assigned to the active arm in the k^th^ trial component. Denote corresponding parameter estimates to be

A chi-square test with 5 degrees of freedom is used to test the hypothesis that

Specifically, we reject the null hypothesis if

, where ,is the lower 95th percentile of chi-square distribution with 5 degrees of freedom, and t(.) denotes vector transpose.

As shown in [23], is asymptotically independent of ; thus, the off-diagonal elements in the first row and first column of will all be zero and

.

Note that can be obtained from the logistic model fitting in (0), and we estimateusing a sandwich type estimator.

For subject i in the case-control sample, let U_1i_, U_2i_, U_3i_, U_4i_ be the score function for models 1 to 4, respectively; if subject i is a case in trial component k, otherwise U_ki_ = (0,0)^T^.

Let I_1_, I_2_, I_3_, I_4_ be the information matrix for models 1 to 4, respectively, with dimensions of 4x4 each, and let N be the total sample size of the case-control sample:

where

if subject i is a case in trial component k, otherwise:

Then the sandwich estimator for variance of can be computed as:

from which we can obtain estimate of .

Furthermore, note that since subjects in the E-alone trial and the E+P trial are non-overlapping, we have , and the covariance term between and in the estimate of is set equal to zero.
