# Supplementary material for: Genetic variants in the MRPS30 region and postmenopausal breast cancer risk
Source: Genome Med. 2011 Jun 24;3(6):42. doi: 10.1186/gm258 (PMC3218816; doi:10.1186/gm258)
Supplement: Additional file 2 — Table S1. Odds ratios for four clinical trial interventions by genotype of rs7705343 in the MRPS30 region according to tumor receptor status. [file gm258-S2.DOC]

**ADDITIONAL FILE 1**

**Table S1.** Odds Ratios for four clinical trial interventions by genotype of rs7705343 in *MRPS30* region, according to tumor receptor status.

|  |  | |  | |  | | Number of Minor Alleles | | | | | |  | |  | |  | |  | |  | |  |
| --- | --- | --- | --- | --- | --- | --- | --- | --- | --- | --- | --- | --- | --- | --- | --- | --- | --- | --- | --- | --- | --- | --- | --- |
|  |  | | 0 | |  | |  | | 1 | |  | |  | | 2 | |  | |  | |  | |  |
|  | OR^†^ Est | | 95% CI | | | | OR^†^ Est | | 95% CI | | | | OR^†^ Est | | 95% CI | | | | *P-*value^‡^ | | Number of Cases | |  |
|  | |  | |  | |  | | **E-alone*** | | | | | |  | |  | |  | |  | |  | |
| ER+ | | 0.460 | | (0.264, | | 0.802) | | 0.956 | | (0.631, | | 1.448) | | 0.949 | | (0.446, | | 2.020) | | 0.091 | | 176 | |
| ER- | | 0.292 | | (0.096, | | 0.887) | | 1.534 | | (0.627, | | 3.752) | | 1.022 | | (0.064, | | 16.35) | | 0.055 | | 40 | |
| PR+ | | 0.606 | | (0.326, | | 1.124) | | 0.912 | | (0.570, | | 1.458) | | 1.136 | | (0.462, | | 2.796) | | 0.441 | | 134 | |
| PR- | | 0.164 | | (0.057, | | 0.470) | | 1.406 | | (0.738, | | 2.677) | | 0.818 | | (0.220, | | 3.046) | | 0.001 | | 76 | |
|  | |  | |  | |  | | **E+P*** | | | | | |  | |  | |  | |  | |  | |
| ER+ | | 1.371 | | (0.920, | | 2.042) | | 1.39 | | (1.034, | | 1.869) | | 1.25 | | (0.789, | | 1.980) | | 0.928 | | 358 | |
| ER- | | 1.715 | | (0.791, | | 3.714) | | 1.349 | | (0.644, | | 2.825) | | 1.905 | | (0.349, | | 10.40) | | 0.879 | | 63 | |
| PR+ | | 1.197 | | (0.768, | | 1.867) | | 1.461 | | (1.055, | | 2.022) | | 1.293 | | (0.793, | | 2.106) | | 0.765 | | 299 | |
| PR- | | 1.905 | | (1.025, | | 3.541) | | 1.381 | | (0.781, | | 2.441) | | 1.27 | | (0.441, | | 3.660) | | 0.696 | | 108 | |
|  | |  | |  | |  | | **DMQ*** | | | | | |  | |  | |  | |  | |  | |
| ER+ | | 0.468 | | (0.296, | | 0.739) | | 0.859 | | (0.624, | | 1.183) | | 1.016 | | (0.584, | | 1.768) | | 0.045 | | 315 | |
| ER- | | 0.643 | | (0.294, | | 1.404) | | 0.750 | | (0.351, | | 1.602) | | 0.833 | | (0.279, | | 2.487) | | 0.923 | | 74 | |
| PR+ | | 0.492 | | (0.301, | | 0.806) | | 0.929 | | (0.657, | | 1.312) | | 0.923 | | (0.495, | | 1.721) | | 0.090 | | 263 | |
| PR- | | 0.485 | | (0.246, | | 0.958) | | 0.730 | | (0.415, | | 1.282) | | 0.964 | | (0.417, | | 2.228) | | 0.427 | | 123 | |
|  | |  | |  | |  | | **CaD*** | | | | | |  | |  | |  | |  | |  | |
| ER+ | | 0.769 | | (0.596, | | 0.992) | | 1.149 | | (0.946, | | 1.396) | | 1.130 | | (0.814, | | 1.569) | | 0.037 | | 796 | |
| ER- | | 0.664 | | (0.387, | | 1.139) | | 0.908 | | (0.558, | | 1.478) | | 0.809 | | (0.389, | | 1.683) | | 0.698 | | 149 | |
| PR+ | | 0.719 | | (0.538, | | 0.963) | | 1.168 | | (0.940, | | 1.451) | | 0.948 | | (0.664, | | 1.354) | | 0.031 | | 640 | |
| PR- | | 0.739 | | (0.498, | | 1.096) | | 1.075 | | (0.763, | | 1.515) | | 1.186 | | (0.664, | | 2.118) | | 0.266 | | 278 | |

^†^OR – estimated intervention odds ratio

^‡^*P-value* – significance level for SNP interaction with CT interventions

*E-alone, estrogen-alone; E+P, estrogen plus progestin; DMQ, low-fat dietary pattern among women having baseline percent of energy from fat in the upper quartile; CaD, calcium plus vitamin D.
