# Supplementary material for: Genetic variants in the MRPS30 region and postmenopausal breast cancer risk
Source: Genome Med. 2011 Jun 24;3(6):42. doi: 10.1186/gm258 (PMC3218816; doi:10.1186/gm258)
Supplement: Additional file 3 — Table S2. Significance levels (P-values) for testing interaction with WHI trial interventions among women with European ancestry for SNPs in the MRPS30 region. [file gm258-S3.DOC]

**ADDITIONAL FILE 2**

**Table S2.** Significance levels (*P-value*s) for testing interaction with WHI trial interventions among women with European ancestry, for SNPs in *MRPS30* region.

| Rs#^*^ | Chromosome | Position | Minor/Major  Allele | E-alone^‡^ | E+P^‡^ | DMQ^‡^ | CaD^‡^ |
| --- | --- | --- | --- | --- | --- | --- | --- |
| 7705343 | 5p12 | 44915334 | G/A | 0.144 | 0.659 | 0.054 | 0.012 |
| 13159598 | 5p12 | 44841683 | G/A | 0.144 | 0.539 | 0.054 | 0.014 |
| 11746980 | 5p12 | 44813635 | A/G | 0.129 | 0.528 | 0.056 | 0.023 |
| 9790879 | 5p12 | 44935642 | C/T | 0.144 | 0.505 | 0.053 | 0.016 |
| 2330572 | 5p12 | 44776746 | C/A | 0.088 | 0.518 | 0.056 | 0.033 |
| 4415084 | 5p12 | 44698272 | T/C | 0.127 | 0.486 | 0.135 | 0.077 |
| 994793 | 5p12 | 44779004 | G/A | 0.129 | 0.482 | 0.056 | 0.023 |
| 2218080 | 5p12 | 44750087 | C/T | 0.227 | 0.793 | 0.049 | 0.032 |

*Rs# – SNP identification (rs) number in dbSNP database;

^‡^E-alone – *P-*value for dependence (interaction) of E-alone odds ratio on SNP from case-only analyses. E+P and CaD are corresponding interaction *P-*values for the other interventions, DMQ is interaction *P-*value for DM among women with baseline % energy from fat in the upper quartile.
