# Supplementary material for: Genetic variants in the MRPS30 region and postmenopausal breast cancer risk
Source: Genome Med. 2011 Jun 24;3(6):42. doi: 10.1186/gm258 (PMC3218816; doi:10.1186/gm258)
Supplement: Additional file 4 — Table S3. Breast cancer odds ratio for WHI trial interventions among women of European ancestry by genotype of the MRPS30 SNP rs7705343. [file gm258-S4.DOC]

**ADDITIONAL FILE 3**

**Table S3.** Breast cancer odds ratio for WHI trial interventions among women of European ancestry, by genotype of *MRPS30* SNP rs7705343.

|  |  |  |  | SNP Genotype | | | | |  |  |  |
| --- | --- | --- | --- | --- | --- | --- | --- | --- | --- | --- | --- |
|  |  |  | GG |  |  | GA |  |  | AA |  |  |
| Intervention | # of Cases | OR^†^ Est | 95% CI | | OR^†^ Est | 95% CI | | OR^†^ Est | 95% CI | | *P*-value^‡^ |
| E-alone | 196 | 0.533 | (0.326, | 0.874) | 0.960 | (0.642, | 1.435) | 1.022 | (0.487, | 2.145) | 0.144 |
| E+P | 415 | 1.358 | (0.959, | 1.923) | 1.178 | (0.896, | 1.548) | 1.478 | (0.927, | 2.357) | 0.659 |
| DMQ | 361 | 0.489 | (0.324, | 0.739) | 0.813 | (0.601, | 1.099) | 1.031 | (0.599, | 1.775) | 0.054 |
| CaD | 921 | 0.693 | (0.549, | 0.876) | 1.081 | (0.901, | 1.297) | 0.960 | (0.705, | 1.307) | 0.012 |

^†^OR – estimated intervention odds ratio

^‡^*P-*value – significance level for SNP interaction with CT intervention
